# Supplementary material for: Air Quality, Pollution and Sustainability Trends in South Asia: A Population-Based Study
Source: Int J Environ Res Public Health. 2022 Jun 20;19(12):7534. doi: 10.3390/ijerph19127534 (PMC9224398; doi:10.3390/ijerph19127534)
Supplement: Supplementary file 1 [file ijerph-19-07534-s001.zip › ijerph-1750109-supplementary.pdf]

Supplementary Materials

Supplementary Table S1: Air Quality Data

Country	Year	Carbon Dioxide	Total Greenhouse Gases	Methane	Nitrous Oxide
Sri Lanka	1990	3.831	29.15	18.41	2.25
Sri Lanka	1991	4.138	29.1	18.24	2.22
Sri Lanka	1992	5.106	30.95	18.72	2.33
Sri Lanka	1993	4.975	31.4	19.18	2.47
Sri Lanka	1994	5.435	32.32	19.29	2.59
Sri Lanka	1995	5.814	32.47	19.48	2.5
Sri Lanka	1996	6.971	32.19	16.73	2.41
Sri Lanka	1997	7.52	30.06	14.6	2.41
Sri Lanka	1998	7.736	27.46	11.75	2.38
Sri Lanka	1999	8.517	26.75	9.98	2.53
Sri Lanka	2000	10.134	25.84	7.71	2.45
Sri Lanka	2001	10.326	23.54	7.61	2.44
Sri Lanka	2002	10.948	24.54	7.84	2.67
Sri Lanka	2003	10.953	25.78	8.23	2.47
Sri Lanka	2004	12.147	25.98	7.89	2.54
Sri Lanka	2005	11.935	27.96	8.56	2.72
Sri Lanka	2006	11.793	26.41	8.61	2.68
Sri Lanka	2007	12.152	27.37	8.46	2.59
Sri Lanka	2008	11.987	27.67	9.16	2.95
Sri Lanka	2009	12.945	26.82	8.98	2.78
Sri Lanka	2010	13.051	28.05	9.45	2.73
Sri Lanka	2011	14.886	29.14	9.64	3.01
Sri Lanka	2012	15.798	30.51	9.76	2.78
Sri Lanka	2013	14.679	28.37	10.09	2.59
Sri Lanka	2014	17.691	31.22	9.22	3.11
Sri Lanka	2015	19.83	34.51	9.65	3.14
Sri Lanka	2016	23.027	37.21	9.9	2.52
Sri Lanka	2017	23.03	37.83	9.11	2.35
Sri Lanka	2018	20.998	37.15	10.03	2.24
Sri Lanka	2019	22.825			
Sri Lanka	2020	21.106			
Pakistan	1990	67.827	166.5	70.35	26.85

Pakistan	1991	67.477	167.45	71.67	27.2
Pakistan	1992	72.015	176.65	72.64	28.79
Pakistan	1993	77.176	186.47	74.85	29.55
Pakistan	1994	84.018	193.14	76.11	32.43
Pakistan	1995	83.614	203.61	77.77	34.74
Pakistan	1996	93.539	210.65	81.42	34.88
Pakistan	1997	93.788	217.56	83.56	36.01
Pakistan	1998	96.741	221.37	85.89	36.5
Pakistan	1999	99.389	233.94	88.41	37.81
Pakistan	2000	105.42	236.06	89.83	38.73
Pakistan	2001	107.126	238.3	91.1	39.13
Pakistan	2002	112.904	244.71	93.78	40.27
Pakistan	2003	117.491	252.54	97.04	41.38
Pakistan	2004	129.96	269.49	99.59	42.68
Pakistan	2005	134.757	277.77	102.46	44.97
Pakistan	2006	143.726	297.16	109.3	46.47
Pakistan	2007	155.846	313.94	112.12	46.43
Pakistan	2008	155.356	316.9	116.86	48.51
Pakistan	2009	155.221	327.27	119.81	52.73
Pakistan	2010	154.145	321.54	119.25	51.84
Pakistan	2011	154.66	328.63	125.32	52.79
Pakistan	2012	153.876	332.43	127.77	51.37
Pakistan	2013	150.451	342.88	132.76	54.81
Pakistan	2014	156.243	356.49	136.69	55.27
Pakistan	2015	169.252	371.24	139.81	56.3
Pakistan	2016	207.019	396.4	143.28	57.87
Pakistan	2017	233.978	421.6	147.54	60.08
Pakistan	2018	238.306	438.22	151.02	60.95
Pakistan	2019	234.289			
Pakistan	2020	234.755			
Bangladesh	1990	14.082	115.06	64.43	16.46
Bangladesh	1991	14.742	115.24	64.51	17.25
Bangladesh	1992	15.38	117.14	65.1	17.55
Bangladesh	1993	16.063	118.35	65.18	17.93
Bangladesh	1994	17.538	120.8	65.99	18.58
Bangladesh	1995	21.042	125.86	66.72	19.86
Bangladesh	1996	21.493	126.39	66.41	20.39
Bangladesh	1997	23.126	128.66	66.99	19.91

Bangladesh	1998	23.198	128.46	66.53	19.95
Bangladesh	1999	25.075	132.37	68.44	21.22
Bangladesh	2000	26.525	134.54	68.99	21.38
Bangladesh	2001	31.035	139.71	69.02	21.91
Bangladesh	2002	31.981	143.03	70.23	22.4
Bangladesh	2003	33.459	144.67	71.08	21.97
Bangladesh	2004	35.945	146.24	70.74	22.07
Bangladesh	2005	37.677	150.91	72.39	22.87
Bangladesh	2006	41.689	156.42	73.44	24.05
Bangladesh	2007	42.642	159.06	73.96	23.98
Bangladesh	2008	45.301	167.36	76.48	26.1
Bangladesh	2009	49.148	171.06	77.4	25.62
Bangladesh	2010	53.992	178.5	78.47	26.08
Bangladesh	2011	56.556	183.76	79.17	27.08
Bangladesh	2012	60.691	187.33	79.58	26.5
Bangladesh	2013	61.782	190.85	80.1	26.78
Bangladesh	2014	65.979	195.62	80.65	27.66
Bangladesh	2015	73.277	204.52	80.92	28.23
Bangladesh	2016	76.477	205.26	80.24	27.28
Bangladesh	2017	80.944	214.18	82.6	28.4
Bangladesh	2018	82.6	220.75	83.81	29.27
Bangladesh	2019	94.215			
Bangladesh	2020	92.842			
India	1990	578.518	1009.44	524.84	141.64
India	1991	615.925	1064.49	530.63	145.7
India	1992	656.033	1096.71	535.41	149.88
India	1993	677.916	1128.44	541.1	153.36
India	1994	716.918	1174.78	545.99	158.98
India	1995	762.121	1240.44	553.59	163.51
India	1996	825.919	1289.63	561.99	167.85
India	1997	859.686	1348.09	568.53	174.13
India	1998	877.698	1382.26	578.38	179.26
India	1999	951.749	1460.24	586.74	183.78
India	2000	978.919	1498.2	590.33	181.23
India	2001	992.56	1747.49	595.27	185.61
India	2002	1023.027	1769.79	589.81	179.55
India	2003	1059.616	1817.42	600.2	188.25
India	2004	1125.471	1905.13	609.1	195.6

India	2005	1185.953	1977.76	622.72	203.88
India	2006	1259.744	2073.67	632.14	213.25
India	2007	1358.152	2219.62	645.57	223.31
India	2008	1462.815	2317.48	651.97	226.38
India	2009	1612.817	2467.78	648.99	234.38
India	2010	1677.888	2576.93	653.89	241.24
India	2011	1780.13	2610.32	655.48	246.58
India	2012	1963.586	2758.53	654.89	246.83
India	2013	2036.937	2816.49	656.08	244.59
India	2014	2185.856	2988.34	655.78	245.91
India	2015	2268.567	3002.17	653.61	247.78
India	2016	2382.223	3073.24	659.81	247.44
India	2017	2433.856	3202.82	665.04	252.04
India	2018	2599.806	3346.63	669.34	256.88
India	2019	2625.968			
India	2020	2441.792			
Bhutan	1990	0.128	-5.46	0.85	0.18
Bhutan	1991	0.175	-5.41	0.85	0.17
Bhutan	1992	0.205	-5.42	0.82	0.17
Bhutan	1993	0.172	-5.45	0.82	0.17
Bhutan	1994	0.202	-5.42	0.82	0.17
Bhutan	1995	0.236	-5.32	0.87	0.18
Bhutan	1996	0.284	-5.29	0.86	0.17
Bhutan	1997	0.375	-5.2	0.87	0.18
Bhutan	1998	0.368	-5.19	0.88	0.18
Bhutan	1999	0.372	-5.13	0.92	0.19
Bhutan	2000	0.383	-5.18	0.88	0.17
Bhutan	2001	0.369	-5.24	0.83	0.17
Bhutan	2002	0.401	-5.21	0.83	0.17
Bhutan	2003	0.36	-5.25	0.83	0.17
Bhutan	2004	0.291	-5.28	0.84	0.18
Bhutan	2005	0.378	-5.09	0.91	0.19
Bhutan	2006	0.374	-5.11	0.91	0.19
Bhutan	2007	0.373	-5.03	0.95	0.22
Bhutan	2008	0.401	-5.16	0.86	0.18
Bhutan	2009	0.369	-5.14	0.9	0.19
Bhutan	2010	0.465	-4.99	0.93	0.21
Bhutan	2011	0.667	0.48	0.91	0.19

Bhutan	2012	0.773	0.62	0.94	0.22
Bhutan	2013	0.853	0.67	0.92	0.19
Bhutan	2014	0.948	0.74	0.9	0.2
Bhutan	2015	0.965	0.77	0.89	0.2
Bhutan	2016	1.156	1.01	0.88	0.19
Bhutan	2017	1.239	1.11	0.87	0.19
Bhutan	2018	1.436	1.16	0.86	0.19
Bhutan	2019	1.927			
Bhutan	2020	1.925			
Nepal	1990	0.721	20.6	16.95	4.48
Nepal	1991	1.026	20.99	17.04	4.54
Nepal	1992	1.233	20.85	16.83	4.55
Nepal	1993	1.397	21.8	17.52	4.64
Nepal	1994	1.619	22.89	17.89	4.82
Nepal	1995	2.309	24.1	18.8	5
Nepal	1996	2.353	24.77	19.46	4.95
Nepal	1997	2.641	25.68	20.03	5.06
Nepal	1998	2.223	26.84	20.81	5.29
Nepal	1999	3.191	28.75	21.59	5.34
Nepal	2000	3.037	29.46	22.15	5.37
Nepal	2001	3.236	27.11	22.27	5.5
Nepal	2002	2.596	26.4	22.5	5.23
Nepal	2003	2.81	27.65	23.21	5.54
Nepal	2004	2.582	27.86	23.6	5.57
Nepal	2005	2.987	28.68	24	5.63
Nepal	2006	2.456	28.47	24.3	5.68
Nepal	2007	2.57	28.8	24.48	5.75
Nepal	2008	3.352	30.37	25.37	6.13
Nepal	2009	4.129	32.45	26.09	6.61
Nepal	2010	4.824	33.93	26.46	6.95
Nepal	2011	5.22	42.54	27.2	7.09
Nepal	2012	5.47	44.84	28.31	7.47
Nepal	2013	6.218	45.78	28.83	7.81
Nepal	2014	7.591	47.78	29.5	8.08
Nepal	2015	6.817	46.82	29.49	7.8
Nepal	2016	9.76	52.26	30.79	9.1
Nepal	2017	11.648	53.06	30.77	8.24
Nepal	2018	13.69	54.57	30.99	8.53

Nepal	2019	16.967			
Nepal	2020	16.958			

Supplementary Table 2: Air Pollution Data

Series Name	Country	1990	2000	2012	2013	2014	2015	2016	2017
PM2.5 air pollution, mean annual exposure (micrograms per cubic meter)	Pakistan	60.334659	61.1060275	62.3766463	61.0221629	59.5183627	60.0858929	58.6327639	58.2824188
PM2.5 air pollution, mean annual exposure	India	81.2906306	84.1648062	88.1694406	91.8047916	89.6223827	89.3033343	89.6721769	90.8732105

(micrograms per cubic meter)									
PM2.5 air pollution, mean annual exposure (micrograms per cubic meter)	Bangladesh	61.5956296	63.0363418	70.7939088	65.6450829	68.3953774	66.9657492	60.1262747	60.8457846
PM2.5 air pollution, mean annual exposure (micrograms per cubic meter)	Bhutan	40.2059691	39.7495788	42.9322729	41.881907	40.8396737	39.8186387	37.2243639	37.9265032

PM2.5 air pollution, mean annual exposure (micrograms per cubic meter)	Nepal	87.5984548	88.9430217	96.9632915	95.3139856	98.1160166	96.2527682	98.054714	99.7343737
PM2.5 air pollution, mean annual exposure (micrograms per cubic meter)	Sri Lanka	29.7685035	30.8557618	30.0839728	28.8982277	26.9227765	25.3373437	14.0254452	11.0996172

Supplementary Table 3: Sustainability Trends Data

	**People using safely managed drinking water services**	**People using safely managed sanitation services**	**Access to electricity**	**Renewable energy consumption**	**Nationally protected terrestrial and marine areas**
	**% of population**	**% of population**	**% of population**	**% of total final energy consumption**	**% of total territorial area**
	**2020**	**2020**	**2017**	**2015**	**2018**
Bangladesh	58.5	38.7	88	34.2	4.9
Bhutan	36.6	65.2	97.7	86.7	48
India	NR	45.9	92.5	34.4	3.5
Nepal	17.6	48.6	89.9	85	23.6
Pakistan	35.8	NR	70.8	45.9	9.8
Sri Lanka	NR	NR	97.5	52.9	3.4
High income	97.7	87	100	11.4	19.1
